# Expression of Concern: Effects of microRNA-146a on the proliferation and apoptosis of human osteoarthritis chondrocytes by targeting *TRAF6* through the NF-κB signaling pathway

**DOI:** 10.1042/BSR-20160578_EOC

**Published:** 2020-09-28

**Keywords:** Chondrocyte, MicroRNA-146a, NF-κB signaling pathway, Osteoarthritis, Proliferation, TRAF6

The Editorial Office has been made aware of potential issues surrounding the scientific validity of this paper, hence has issued an expression of concern to notify readers whilst the Editorial Office investigates.

